# Supplementary material for: Ticagrelor Versus Clopidogrel in Older Patients with NSTE-ACS Using Oral Anticoagulation: A Sub-Analysis of the POPular Age Trial
Source: J Clin Med. 2020 Oct 12;9(10):3249. doi: 10.3390/jcm9103249 (PMC7601891; doi:10.3390/jcm9103249)
Supplement: Supplementary file 1 [file jcm-09-03249-s001.pdf]

Supplementary materials

Table S1. Reasons to discontinue the P2Y12 inhibitor

	**OAC + Clopidogrel**<br>**N= 22**	**OAC + Ticagrelor**<br>**N= 74**
Bleeding	2 (9.1)	9 (12.2)
OAC	5 (22.7)	26 (35.1)
Dyspnea	0	11 (14.9)
Revision of diagnosis	9 (40.9)	8 (10.8)
CABG	1 (4.5)	12 (16.2)
Side-effect	0	4 (5.4)
Other/unknown	5 (22.7)	4 (5.4)

Data are n (%). OAC start of oral anticoagulation; revision of diagnosis i.e. type II ischemia, no significant stenosis on coronary angiography; CABG undergoing coronary artery bypass grafting during hospital stay; side effect other than bleeding or dyspnoea.

Table S2. Bleeding outcomes at 30 days

	**OAC + Clopidogrel N= 83**	**OAC + Ticagrelor N= 101**	**HR (95% CI)**	**P-value**
PLATO major and minor bleeding	6 (7.3)	17 (16.9)	0.38 (0.15-0.99)	0.052
PLATO minor bleeding	1 (1.2)	6 (6.0)	0.23 (0.03-1.93)	0.098
PLATO other major bleeding	4 (4.9)	6 (6.1)	0.71 (0.20-2.60)	0.735
PLATO major life threatening bleeding	2 (2.4)	5 (5.0)	0.44 (0.08-2.37)	0.359

PLATO major bleeding	5 (6.1)	11 (11.1)	0.47 (0.16-1.38)	0.233
PLATO non-CABG related major bleeding	3 (3.7)	6 (6.1)	0.55 (0.13-2.26)	0.460
ICH	0	0	-	-
Fatal bleeding	0	(1.0)	-	0.363

Data are n (%). CABG: coronary artery bypass grafting; CI: confidence interval; HR: hazard ratio; ICH: intracranial haemorrhage; OAC: oral anticoagulation; PLATO: PLATelet inhibition and patient Outcomes.

Table S3. Primary net clinical benefit outcome and secondary thrombotic outcomes at 30 days

	**OAC + Clopidogrel N= 83**	**OAC + Ticagrelor N= 101**	**HR (95% CI)**	**p-value**
Net clinical benefit outcome				
All-cause death, myocardial infarction, stroke, PLATO major and minor bleeding	8 (9.6)	26 (25.7)	0.34 (0.15-0.75)	0.005
Second net clinical benefit outcome				
CV death, myocardial infarction, stroke, PLATO major bleeding	7 (8.4)	21 (20.8)	0.35 (0.15-0.84)	0.019
Thrombotic outcomes				
CV death, myocardial infarction, stroke	2 (2.4)	11 (11.0)	0.22 (0.05-1.00)	0.025
All-cause death	1 (1.2)	6 (5.9)	0.19 (0.02-1.64)	0.098

CV death	1 (1.2)	4 (4.0)	0.32 (0.04-2.94)	0.255
Myocardial infarction	1 (1.2)	5 (5.0)	0.23 (0.03-1.99)	0.151
Unstable Angina	0	0	-	-
Ischemic stroke	0	3 (3.0)	-	0.112
Transient ischemic attack	0	0	-	-
Stent thrombosis	0	0	-	-
Urgent revascularization	0	1 (1.0)	-	0.355

Data are n (%). CI: confidence interval; CV: cardiovascular; HR: hazard ratio; OAC: oral anticoagulation; PLATO; PLATelet inhibition and patient Outcomes.

Table S4. Per protocol analysis

	**OAC + Clopidogrel N= 83**	**OAC + Ticagrelor N= 101**	**HR (95% CI)**	**P-value**
PLATO major and minor bleeding	16 (23.6)	22 (42.6)	0.46 [0.24-0.88]	0.016
All-cause death, myocardial infarction, stroke, PLATO major and minor bleeding	20 (28.9)	31 (59.8)	0.37 [0.21-0.65]	<0.001
CV death, myocardial infarction, stroke	5 (7.4)	9 (19.0)	0.34 [0.11-1.04]	0.049

Data are n (%). CI: confidence interval; CV: cardiovascular; HR: hazard ratio; OAC: oral anticoagulation; PLATO; PLATelet inhibition and patient Outcomes.

Table S5. Sensitivity analysis of patient using OAC at moment of randomization

	OAC + Clopidogrel N= 56	OAC + Ticagrelor N= 64	HR (95% CI)	P-value
PLATO major and minor bleeding	7 (13.0)	25 (40.6)	0.25 (0.11-0.59)	0.001
All-cause death, myocardial infarction, stroke, PLATO major and minor bleeding	13 (23.2)	34 (53.1)	0.34 (0.18-0.65)	<0.001
CV death, myocardial infarction, stroke	7 (12.5)	10 (16.5)	0.81 (0.31-2.13)	0.508

Data are n (%). CI: confidence interval; CV: cardiovascular; HR: hazard ratio; OAC: oral anticoagulation; PLATO; PLATelet inhibition and patient Outcomes.

Table S6. Sensitivity analysis of patient using OAC and a P2Y12 inhibitor at moment of randomization

	OAC + Clopidogrel N= 68	OAC + Ticagrelor N= 52	HR (95% CI)	P-value
PLATO major and minor bleeding	11 (16.7)	17 (34.1)	0.39 (0.18-0.85)	0.021
All-cause death, myocardial infarction, stroke, PLATO major and minor bleeding	17 (0.25)	24 (46.2)	0.45 (0.24-0.84)	0.009
CV death, myocardial infarction, stroke	7 (10.3)	8 (15.8)	0.75 (0.27-2.08)	0.344

Data are n (%). CI: confidence interval; CV: cardiovascular; HR: hazard ratio; OAC: oral anticoagulation; PLATO; PLATelet inhibition and patient Outcomes.

Table S7. Multivariable analysis. Propensity score weighted Cox proportional hazard regression.

	**HR (95% CI)**	**P-value**
PLATO major and minor bleeding	0.58 (0.30-1.11)	0.100
All-cause death, myocardial infarction, stroke, PLATO major and minor bleeding	0.51 (0.30-0.89)	0.020

Data are n (%). CI: confidence interval; CV: cardiovascular; HR: hazard ratio; OAC: oral anticoagulation; PLATO; PLATelet inhibition and patient Outcomes.
